# Outline and validation of a new dispatcher-assisted cardiopulmonary resuscitation educational bundle using the Delphi method

**DOI:** 10.1016/j.resplu.2023.100542

**Published:** 2024-01-04

**Authors:** Andreas Claesson, Håkan Hult, Gabriel Riva, Fredrik Byrsell, Thomas Hermansson, Leif Svensson, Therese Djärv, Mattias Ringh, Per Nordberg, Martin Jonsson, Sune Forsberg, Jacob Hollenberg, Anette Nord

**Affiliations:** a Center for Resuscitation Science, Department of Clinical Science and Education, Karolinska Institutet, Södersjukhuset, Stockholm, Sweden; b Department of Healthcare, Clinicum, Linköping University Hospital, Sweden; c Department of Medicine Solna, Karolinska Institutet, Stockholm, Sweden

**Keywords:** Out-of-hospital cardiac arrest (OHCA), Emergency medical dispatch centre (EMDC), Dispatcher, Cardiopulmonary resuscitation (CPR), CPR training, DA-CPR, Education, Emergency calls

## Abstract

**Aim:**

Dispatcher-assisted cardiopulmonary resuscitation (DA-CPR) is time-dependent. To date, evidence-based training programmes for dispatchers are lacking. This study aimed to reach expert consensus on an educational bundle content for dispatchers to provide DA-CPR using the Delphi method.

**Method:**

An educational bundle was created by the Swedish Resuscitation Council consisting of three parts: e-learning on DA-CPR, basic life support training and audit of emergency out-of-hospital cardiac arrest calls. Thereafter, a two-round modified Delphi study was conducted between November 2022 and March 2023; 37 experts with broad clinical and/or scientific knowledge of DA-CPR were invited. In the first round, the experts participated in the e-learning module and answered a questionnaire with 13 closed and open questions, whereafter the e-learning part of the bundle was revised. In the second round, the revised e-learning part was evaluated using Likert scores (20 items). The predefined consensus level was set at 80%.

**Results:**

Delphi rounds one and two were assessed by 20 and 18 of the invited experts, respectively. In round one, 18 experts (18 of 20, 90%) stated that they did not miss any content in the programme. In round two, the scale-level content validity index based on the average method (S-CVI/AVE, 0.99) and scale-level content validity index based on universal agreement (S-CVI/UA, 0.85) exceeded the threshold level of 80%.

**Conclusion:**

Expert consensus on the educational bundle content was reached using the Delphi method. Further work is required to evaluate its effect in real-world out-of-hospital cardiac arrest calls.

## Introduction

Early recognition of cardiac arrest by dispatchers is time-critical. Each minute of delay in cardiopulmonary resuscitation (CPR) and defibrillation reduces the chance of survival.^1^ The emergency medical dispatch centre (EMDC) plays an essential role in the chain of survival,^2^, ^3^ and high-performing EDMCs may have an impact on subsequent interventions. Early recognition of out-of-hospital cardiac arrest (OHCA) by medical dispatchers may thereby lead to (a) earlier activation of emergency medical services in parallel with (b) earlier initiation of dispatch-assisted CPR (DA-CPR) and (c) earlier referral of lay responders to a nearby automated external defibrillator (AED).

Recognition of OHCA during an emergency call is a challenging task, and dispatcher recognition of OHCA during emergency calls varies between 46% and 98%.^3^, ^4^, ^5^, ^6^, ^7^ Kurz et al.^2^ indicate that thousands of additional lives could be saved annually worldwide if the American Heart Association’s (AHA) goals for DA-CPR were met. A study by Byrsell et al.^6^ suggests that if the EMDC further optimizes the handling of OHCA calls in accordance with the AHA high-performance goals for DA-CPR, more than 400 additional lives could be saved annually in Sweden. A combination of medical science, educational efficacy and local implementation are required and essential for improved survival.^8^

Several studies have shown that different training interventions at the EMDC have beneficial effects on dispatchers’ performance in OHCA call handling. Training can increase the proportion of cardiac arrest calls recognized and decrease the time to first DA-CPR compressions.^9^, ^10^, ^11^, ^12^, ^13^ However, courses aimed at dispatchers are usually created locally at the EMDC organization and there is a lack of standardized training.^11^ The European Resuscitation Councils (ERC) guidelines indicate that most of the interventions are time-consuming and there is no structured course to train dispatchers.^11^

Essential core skills in dispatchers call management need to be defined and taught to improve patient survival after cardiac arrest.^11^ This study aimed to reach expert consensus on an educational bundle content for dispatchers to provide DA-CPR using the Delphi method.

## Method

### Study design

On behalf of the Swedish Resuscitation Council, six professionals (medical doctors, registered nurse/medical dispatchers, a professor of medical pedagogy and a graphic designer) in the field of OHCA created a new educational bundle called *Dispatch-assisted CPR – a race against the clock*. The content of the educational bundle was based on the ERC guidelines (2021) and the recommendations and performance measures outlined by the AHA and the Resuscitation Academy.^2^, ^3^ The latter part of the development process was based on a modified Delphi technique, focusing on core content for DA-CPR handling and education. In a two-round Delphi study, participating experts completed questionnaires to rate the content of the new educational bundle. In the current study, experts were defined as “individuals with experience in handling OHCA calls” and “specialists in the field”.^14^.

### Content of the new educational bundle

The education bundle consists of three mandatory modules. Most of the training sessions can be carried out by the dispatchers individually when it suits the EMDC both practically and in terms of time.

#### Module 1: E-learning programme

The new e-learning programme consists of information about all parts of the educational bundle, the learning objectives and information about cardiac arrest, recognition and a “no, no, go” approach,^2^ the quality of DA-CPR, how to deal with barriers to DA-CPR, real-life audio files on recognition, agonal breathing, measuring performance of OHCA call handling, example of high-performance managed calls, short videos, interactivity tasks and quizzes.^2^, ^3^ The participant must answer all the questions correctly to pass the test. The e-learning programme takes approximately 30 min to complete and is recommended to be performed every 6 months.

#### Module 2: Practical CPR training on a manikin

All staff at the EMDC are advised to attend an annual practical basic life support (BLS) training course for both adults and children, led by a certified CPR instructor at the EMDC (the only part that is carried out in a group). For quality improvement of CPR skills, low-dose high-frequency training has been shown to be effective.^15^ Each dispatcher is also recommended to test their practical CPR skills on a manikin using a feedback device (Laerdal Medical QCPR Little Anne) during a 2-minute training session once a month. The dispatchers are encouraged to achieve at least 80% correctly performed chest compressions during CPR.

#### Module 3: Auditing and measuring the performance of OHCA calls

Hattie,^16^, ^17^ professor of education, states that feedback is one of the most powerful influences on learning and achievement. Continuous quality improvement and feedback on cardiac arrest calls are recommended according to guidelines.^2^, ^3^, ^11^ All OHCA calls registered in the Swedish Registry of Cardiopulmonary Resuscitation (SRCR, i.e. validated OHCA calls) and handled by the EMDC should be audited retrospectively by the dispatcher who handled the call with individual feedback from a CPR instructor or supervisor at the EMDC. The call management evaluation template is based on AHA performance goals for DA-CPR and a modified version of the Cardiac Arrest Registry to Enhance Survival (CARES) DA-CPR data dictionary with 12 mandatory variables (Table 1).^2^, ^18^ Data should be extracted from the SRCR once a month for timely feedback after a call.

**Table 1:** Summary of call management evaluation template.

*Mandatory variables*
1. Filename
2. CPR already in progress (yes, no, unknown)
3. Consciousness addressed (yes, no, unknown, NA)
4. Abnormal breathing addressed (yes, no, unknown, NA)
5. Did dispatcher recognise need for CPR (yes, no, unknown)
6. Time of recognition (min:sec)
7. Time to dispatch an ambulance (min:sec)
8. CPR instructions started (yes, no, unknown, NA)
9. Time to DA-CPR instruction (min:sec)
10. Time of first chest compression (min:sec)
11. Barriers delaying or hindering CPR?
12. Was an AED addressed (yes, no, unknown, NA)
*Extended review*

13. The dispatcher instructed the caller to activate the speaker phone? (yes, no, unknown, NA)
14. Was caller alone at time of call? (yes, no, unknown)
15. Is the dispatcher assertive or passive when providing CPR instructions? (assertive, passive, NA)
16. The dispatcher checked the CPR quality and used encouragement techniques? (yes, no, NA)
17. Was an AED connected to the patient? (yes, no, unknown, NA)
18. Call continued until EMS arrival? (yes, no, unknown)
19. Other /reflection

AED, automated external defibrillator; CPR, cardiopulmonary resuscitation; DA-CPR, dispatcher-assisted cardiopulmonary resuscitation; EMS, emergency medical services; min:sec, minutes:seconds; NA, not applicable.

#### Delphi validation procedure

In the Delphi method, experts' opinions are obtained through repeated surveys which are answered anonymously.^14^, ^19^, ^20^, ^21^ The Delphi process continues until the experts no longer anticipate further increases in consensus for statements. The overall aim of an expert panel is to identify broad areas of agreement.^19^

**Fig. 1:**
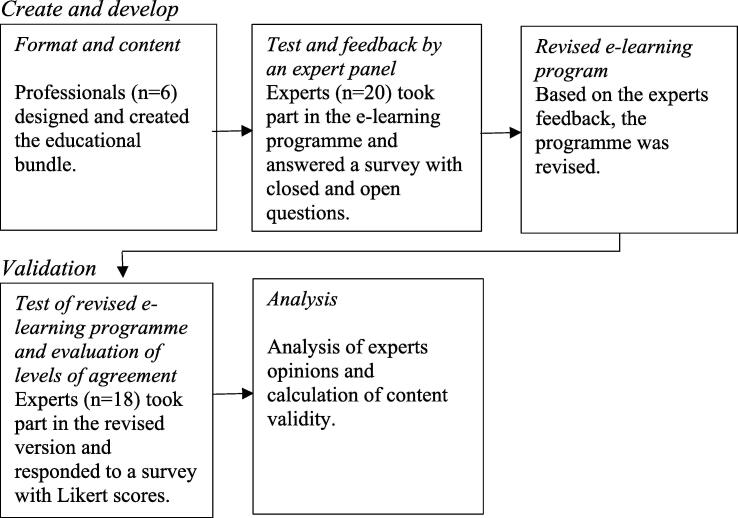
Flowchart of the development and validation process of the educational bundle.

All experts gave written consent. Their participation was voluntary and they could choose to discontinue participation at any time. The study used non-sensitive data and the participants were assured of confidentiality and anonymity within the framework of the study and reports from the study. Participating experts and their answers were blinded to each other throughout the study. The principles stated in the World Medical Association Declaration of Helsinki and the Swedish Ethics Review Board were followed. A summary and flowchart of the creation and validation procedure of the educational bundle are shown in Fig. 1.

#### Round 1: Test and feedback by an expert panel

After the team of professionals had designed and created the educational bundle, links to module 1 (the e-learning part) and an online survey was sent out to 37 experts, none of whom had participated in the development process of the educational bundle. The selection of respondents was made in consultation with senior managers and staff responsible for CPR training at the EMDC to reach experts with broad clinical and/or scientific knowledge of DA-CPR. The inclusion criteria for the expert group were as follows: (a) an active professional in education with experience teaching DA-CPR, (b) a registered nurse or medical dispatcher with clinical experience in handling cardiac arrest calls, or (c) a physician or medical doctor in the field of medicine. The experts worked at various EMDCs in Sweden.

Links to the e-learning part and the online survey was sent by e-mail in November 2022; two reminder e-mails were sent. This first survey with 13 closed and open-ended questions aimed to investigate (a) what the participants considered to be the strengths and weaknesses of the educational bundle, (b) how they perceived the level of content, (c) the time required to complete the e-learning module, and (d) how they experienced navigating the e-learning programme. Although the experts only performed the e-learning (module 1), it includes details of the entire educational bundle (including modules 2 and 3). The experts thus had the opportunity to give their views on modules 2 and 3.

Based on the feedback from the experts in the first round, the educational bundle was revised (version 2.0). An interactive section on putting the parts in the chain of survival together and an animated film about cardiac arrest were removed, an additional audio file on agonal breathing was added, the quality of some of the audio files was updated and some text was reworded to reduce misunderstandings.

#### Round 2: Test of the revised programme and evaluation of the levels of agreement

In the second round (February 2023), the same group of experts were invited by e-mail to take part in a new round using the revised e-learning module (2.0) and answer a survey with 20 closed questions and 1 open question with the possibility of adding individual comments. The aim of the second round was to evaluate if the revised e-learning programme could reach broad areas of agreement within the expert panel.^19^ The content of each chapter and the suitability of the audio files, films, interactive tasks and quizzes were assessed using Likert scores with four response options: very relevant, quite relevant, somewhat relevant or not relevant.^14^, ^19^ A response to each closed question was mandatory to proceed through the survey. Two reminders were sent by e-mail. Based on the results in round 2, content validity was calculated.

### Statistical analysis

• This study used two forms of content validity index (CVI): CVI for item (I-CVI) and CVI for scale (S-CVI). The definition and formula are based on the recommendations described by Yusoff et al.^22^ The four-point Likert scale was dichotomized to calculate the CVI to a two-point scale with “not relevant” and “somewhat relevant” representing “0”, and “quite relevant” and “highly relevant” representing 1.^22^ In the next step, we counted the number of experts who agreed with the content for each item and then registered the points on which the experts reached universal consensus (0 or 1).^14^ Based on these data, the following indices were calculated:^22^
• I-CVI: the proportion of all experts that scored the item as relevant (experts in agreement divided by the number of experts)
• “Scale-level content validity index based on the average method” (S-CVI/AVE): the proportion of all items scored as relevant of all items in the tool (the average of I-CVI scores across all items)
• “Scale-level content validity index based on the universal agreement method” (S-CVI/UA): proportion of items scored as relevant by all experts (average UA scores across all items).^22^

There are no clear guidelines for the level of consensus in the Delphi process, however 75%–80% has been recommended.^20^, ^23^ For this study, according to the research plan, we considered consensus to be achieved when 80% of the experts agreed on the importance of the content. A modified abbreviated version of qualitative inductive content analysis was applied in the analysis of data from the open-ended questions.^24^

## Results

### Round 1: Feedback and development

Thirty-seven experts were invited to participate in the study and 20 (54%) participated in round one. The questionnaire was answered by staff working at any EMDC; 2 physicians/medical doctors, 9 registered nurses, 6 medical dispatchers and 3 from other professions such as manager. All 20 experts (100%) rated the content to be informative and instructive. Eighteen experts (18 of 20, 90%) stated that they did not miss any content/segment in the programme. One expert requested additional audio files of OHCA calls. Another suggested additional advice on how to communicate with the caller during cardiac arrest calls and the order of standardized phrases. Three (3 of 20, 15%) experts suggested content that could be removed from the e-learning module: an interactive section on putting the parts in the chain of survival together and an animated film about cardiac arrest. Four themes emerged from the analysis of the responses on what the experts appreciated most in the educational bundle: (a) audit of real audio files of OHCA calls and agonal breathing, (b) concrete advice on managing and overcoming common barriers to DA-CPR, (c) understanding the importance of early recognition of OHCA, and (d) annual practical hands-on training, i.e. BLS training. Weaknesses, as described by four (4 of 20, 20%) experts, were the quality of some of the audio files, a lot of text to read initially, some minor rewording and some parts of the training were too basic. Experts acknowledged that the basic level was needed for inexperienced dispatchers, however they requested a parallel in-depth part for special circumstances. All experts indicated that it was easy (n = 7, 35%) or very easy (n = 13, 65%) to navigate the e-learning programme.

### Round 2: Content validity

In round two, the questionnaire was answered by 18 experts: 1 medical doctor, 6 nurses, 7 medical dispatchers and 4 from other professions such as manager. All content validity indices (CVIs) are presented in Table 2. Sixteen experts (16 of 18, 89%) agreed that all the items in the educational bundle were highly relevant. One expert considered that the written content in chapter 2, *About cardiac arrest*, was somewhat relevant and that some of the quizzes in the same chapter were not relevant, which resulted in an I-CVI of 0.94 for these two items. Another expert considered that some of the quizzes in chapter 5, quality follow-up of OHCA calls, were somewhat relevant, which resulted in an I-CVI of 0.94. There was universal agreement for all remaining items (n = 17) regarding written content, images, audio files, videos, quizzes and interactivities, resulting in I-CVIs of 1.00. We obtained an S-CVI/AVE of 0.99 and an S-CVI/UA of 0.85 in the validation of the content of the educational bundle.

**Table 2:** Round 2: content validity index values.

Content	Experts in agreement (n = 18)	Universal agreement	I-CVI
Chapter 1. Introduction			
Written content	18	1	1.0
Image	18	1	1.0
Quiz	18	1	1.0
Chapter 2. Cardiac arrest			
Written content	17	0	0.94
Image	18	1	1.0
Quiz and interactivity	17	0	0.94
Chapter 3. Recognize OHCA			
Written content	18	1	1.0
Image	18	1	1.0
Audio files	18	1	1.0
Quiz	18	1	1.0
Chapter 4. High-quality CPR and AED			
Written content	18	1	1.0
Image	18	1	1.0
Audio files	18	1	1.0
Quiz	18	1	1.0
Chapter 5. Quality improvement			
Written content	18	1	1.0
Image	18	1	1.0
Quiz including audio files	17	0	0.94
Chapter 6. Algorithms and videos			
Written content, algorithms	18	1	1.0
Instructional videos	18	1	1.0
Chapter 7. Research			
Written content	18	1	1.0
Summary		17	19.82
S-CVI/AVE (19.82/20)		0.99	
S-CVI/UA (17/20)		0.85	

I-CVI, item-level content validity index; S-CVI, scale-level content validity index based on the average method; S-CVI/UA, scale-level content validity index based on universal agreement.

## Discussion

This study describes the creation and validation process of an educational bundle to enhance DA-CPR using the Delphi method. The content of this new educational bundle, *Dispatch-assisted CPR – a race against the clock,* reached consensus in a Delphi process. We conclude that the S-CVI/AVE value (0.99) and S-CVI/UA value (0.85) exceeded the threshold level of 80% and thus achieved a satisfactory level of content validity.^20^, ^23^

The Delphi method aims to achieve general consensus on a specific topic based on expert opinions collected via repeated surveys.^19^, ^20^, ^21^ The Delphi method has been used previously to evaluate the content of various education programmes.^14^, ^25^, ^26^ A strength of the Delphi method is that all experts have equal influence on the content validity consensus process. The expert panel in the present study represented various professions, with geographic spread, in the field of emergency medical dispatch centres and contributed a variety of relevant points of view to the Delphi process. When collecting data there is a risk of potential social-desirability bias. However, an advantage with the Delphi method is the anonymity of the respondents, which reduces the risk of other group members' opinions or group pressure influencing the respondent's answers.^21^

In the Delphi process, the selection and number of appropriately qualified experts affect the reliability of the study. No clear guidance on the definition of an expert exists in the Delphi literature.^20^ In the present study, experts were defined as “individuals with experience in handling OHCA calls” and “specialists in the field”.^14^ We aimed for a heterogeneous group of experts in terms of professions and experience. The response rate was 54% (n = 20) in round 1 and 48% (n = 18) in round 2. However, all invited professions (physicians, medical doctors, nurses, medical dispatchers and persons from other professions such as managers) were represented in both rounds 1 and 2. In accordance with the Delphi literature, the total number of participating experts is considered sufficient.^20^, ^21^ Although participation took place within the framework of the experts' work, it was time-consuming to participate as an expert in the present study. The experts had to complete the e-learning module (approx. 30 min) and answer the survey, which may have contributed to some refraining from participating.

The content of this new educational bundle is judged to be relevant using the Delphi method, and the experts reached consensus for 17 of 20 items. The three items that did not reach full consensus were mainly quizzes to check the participants’ knowledge. However, how the new educational bundle affects medical dispatchers’ handling of cardiac arrest calls has not yet been evaluated. The next step is to scientifically evaluate the educational bundle using a prospective intervention at the EMDC to assess whether the training affects dispatchers’ real-world handling of cardiac arrest calls in accordance with AHA goals.

## Limitations

Although rounds 1 and 2 included participants from different professions and genders, the number of years in the profession and gender are unknown because the participants were not asked to provide this information to ensure anonymity. Another limitation is the fact that the definition of an expert is subjective.^20^ Our definition of an expert was based on subjective experience within the author group, with extensive research experience at EMDCs, and clearly defined in the research plan. In accordance with several previous studies applying the Delphi method of content validation, the experts were not given the opportunity to provide input during the creation of the initial prototype package. On the other hand, the first online survey contained both closed and open questions, allowing the experts to provide their individual views on both content and design.

The educational bundle consists of three modules, but the expert panel only took part in the e-learning module (module 1). However, the e-learning module describes the other two modules in detail, including supplementary files for quality follow-up of OHCA calls. Thus, the experts are assumed to have assessed and evaluated the content of modules 2 and 3 at least to a theoretical extent. The generalizability can also be affected by the fact that only Swedish experts were included and limited response frequency*.*

## Conclusion

Expert consensus on the educational bundle content was reached using the Delphi method. Further work is required to evaluate its effect in real-world OHCA calls.

## Funding

This study was funded by the 10.13039/501100003793Swedish Heart-Lung Foundation (20210282) and the 10.13039/501100004102Laerdal Foundation (2023-0018). The funders had no role in the design, analysis or writing of the article.

## CRediT authorship contribution statement

**Andreas Claesson:** Conceptualization, Methodology, Writing – review & editing. **Håkan Hult:** Methodology, Writing – review & editing. **Gabriel Riva:** Methodology, Writing – review & editing. **Fredrik Byrsell:** Methodology, Writing – review & editing. **Thomas Hermansson:** Conceptualization, Methodology, Writing – review & editing. **Leif Svensson:** Methodology, Writing – review & editing. **Therese Djärv:** Writing – review & editing. **Mattias Ringh:** Writing – review & editing. **Per Nordberg:** Writing – review & editing. **Martin Jonsson:** Formal analysis, Writing – review & editing. **Sune Forsberg:** Writing – review & editing. **Jacob Hollenberg:** Writing – review & editing. **Anette Nord:** Conceptualization, Data curation, Formal analysis, Methodology, Writing – original draft, Writing – review & editing.

## Declaration of competing interest

The authors declare that they have no known competing financial interests or personal relationships that could have appeared to influence the work reported in this paper.
